# "Sponsors" for Out-Patients

**Published:** 1887-07-30

**Authors:** 


### "SPONSORS" FOR OUT-PATIENTS.

"Sponsors" make one think of catechisms. Mr. George Stoker, who is anxious to introduce the former into the out-patient system, says nothing about the latter. Perhaps he was not very familiar with his catechism as a boy, and has forgotten the answer to the third question in that significant "Principia." Mr. Stoker offers a practical suggestion for the better management of the out-patient department, and in so doing proves himself to be a man of a hopeful disposition. He reminds us of the Israelites who besieged Jericho by the prolonged blowing of trumpets; but although the walls of that famous city fell down after seven days of trumpet-blowing, we would warn Mr. Stoker that that is the only historical example of such a method of successful siege. In later times it has generally happened, as the first Napoleon said, that "Providence has been on the side of the big battalions," so that penny trumpets are, to a large extent, discredited as warlike implements. Don Quixote for a time seemed to be successful in restoring that spirit of chivalry which emboldened a single man to lay lance in rest, and charge valiantly uncounted hosts of enemies. But in the case of that renowned knight it must not be forgotten that though he achieved a signal victory against an innumerable body of sheep, yet even he was compelled to bite the dust when he essayed to overthrow a windmill. We by no means desire to discourage Mr. Stoker in his laudable efforts. A little gunpowder will blow up a very big rock. Is the sponsorial, or as Mr. Stoker prefers to call it, the "voucher" system, of a gunpowdery nature, or is it more allied to Rhubarb or Pulv. Cinnam. Co., and therefore very mild in its physiological results?

Of this we are absolutely convinced, that no walls of Jericho could be thicker or more solid than the dense mass of indifference which surrounds the question of the out-patient department. Roman battering-rams, Cromwellian twelve-pounders, modern Krupps, and every other kind of artillery have been hurled against the system in vain. The defenders look blandly out from their port-holes, and smilingly call upon the besiegers to "fire away." Now we greatly admire serenity of temper, but when it is accompanied by and significant of administrative incapacity, our admiration is largely replaced by other and less amiable feelings. The fact is that the management of the out-patient department at many hospitals is a scandal and a disgrace. People may smile and shrug their shoulders to see it so described, but we do not envy such people either their toughened consciences or their blinded eyes.

By way of a step in a new direction, Mr. Stoker proposes the "voucher" system. This is very simple, but not the less to be taken into consideration on that account. According to it, every person who applies at the out-patient department is to be seen and prescribed for "once" on his personal application alone. When he goes away, a card is handed to him to be filled up by a clergyman or householder before his return. The clergyman or householder is called upon to certify that he knows the patient and considers him to be a fit and proper person to receive charitable aid. In other words, the clergyman or householder, by signing the card or voucher, is to become to that extent sponsor for the intending out-patient. No person is to be seen a second time who does not bring his voucher duly filled up and signed.

This system is not new, although we are not aware that it has been extensively tried in town or country. It has one fatal defect. In allowing any householder to become sponsor for his fellow by filling up and signing the voucher, it simply substitutes one unknown and totally irresponsible person for another—the patient's next-door neighbour for the patient himself—Richard Smith for Robert Jones. Smith will never by any possibility refuse to sign Jones's voucher, for the simple reason that he may want Jones to sign his own next month, next week, or even next day. In the "voucher" system everything depends upon the person who vouches. Even sponsors who solemnly promise in an infant's name to "renounce the devil and all his works, the pomps and vanities of this wicked world, and all the sinful lusts of the flesh," very often fulfil their promises by giving a silver mug, and never speaking to the child from that day onwards. If educated persons will thus readily take upon themselves, with every outward solemnity, such serious responsibilities, and then think no more of them to the end of their days, what importance can it be thought a working-man or small tradesman will attach to the signing of a hospital voucher? If you can get persons to vouch who have adequate knowledge and a due sense of responsibility, persons whose signatures are not appended to such cheques without assets at the bank of conscience, you will accomplish a good deal—perhaps all you want. But where are those persons to be found, and especially to be found when wanted?

The "voucher" system as propounded by Mr. Stoker will not do. Some form of such a system might, however, finally settle the question, because all that is wanted is a reliable assurance that the would-be patient is really a fit object for gratuitous medical relief. The mere householder as a voucher must be peremptorily dismissed! Can a substitute or substitutes be found? We think they can. Four classes of substitutes readily occur to the mind, viz., clergymen and other recognised ministers of religion, medical men, subscribers to the hospital, and duly appointed agents of the Charity Organisation Society. Mr. Stoker indeed objects to medical men by anticipation, but therein he shows a decidedly unjustifiable animus, probably the result of a limited acquaintance with his brother practitioners. Medical men we consider the most suitable of all the classes of sponsors we have named, and that for several reasons. Their knowledge of the subject is of necessity infinitely superior to that of any other class, and their well-known benevolence is a sufficient guarantee of their readiness to recommend suitable cases. For medical men, we maintain, are by training, habit, and instinct more purely benevolent than any other class, if we except the clergy. It is their interest to cultivate benevolence, and that, which perhaps originally is commenced for the sake of self-recommendation, gradually becomes a steady and approved habit of mind and conduct. Anyone who has seen medical practice among the poor, knows well how willing and even anxious the doctor is to hand over a tedious and unsatisfactory case to the hospital. He knows, if he knows nothing else, that a poor man partially or completely disabled, has no means of paying medical fees, and in self-defence, if for no other reason, he is disposed to send him for free relief. But we malign medical men if we suppose any considerable number of them would stand in the way of any patient who really needed the aid which hospitals have to give. They above all men are fit to act as sponsors for the poor and sick who have no resources but such as charity provides. The "voucher" system on some such bases as we have indicated is well worth a thorough and lasting trial, and we venture to recommend it to the sober consideration of intelligent and conscientious hospital managers both in town and country. The "part pay" system, with which Mr. Stoker proposes to associate it, we reserve for consideration at some future time.

